# *Latilactobacillus sakei* strains protect crucian carp against *Aeromonas hydrophila*–induced intestinal injury in an oral challenge model

**DOI:** 10.3389/fnut.2026.1768111

**Published:** 2026-02-05

**Authors:** Yan Zhao, Ruizhe Li, Ting Zhu, Huanhuan Hu, Guanghui An, Zhangheng Ren, Jie Cui, Jinchi Jiang

**Affiliations:** 1 Jiangsu Key Laboratory of Marine Bioresources and Environment, Jiangsu Key Laboratory of Marine Biotechnology, Jiangsu Ocean University, Lianyungang, China; 2 Co-Innovation Center of Jiangsu Marine Bio-industry Technology, Jiangsu Ocean University, Lianyungang, China; 3 Clinical Nutrition Department, Children’s Hospital of Nanjing Medical University, Nanjing, Jiangsu, China; 4 College of Food Science and Light Industry, Nanjing Tech University, Nanjing, China

**Keywords:** *Aeromonas hydrophila*, crucian carp, inflammation, *Latilactobacillus sakei*, transcriptom

## Abstract

In aquaculture, the overuse of antibiotic could lead to antimicrobial resistance and destabilize host–microbiota homeostasis. *Latilactobacillus sakei,* belonging to the genus *Latilactobacillus,* was included in the list of bacteria that could be used in food in China in 2014. Increasing evidence demonstrated that its antagonistic capacity against a broad spectrum of pathogenic bacteria, indicating its promising potential for application in aquaculture. In this study, the protective effect of three *L. sakei* (JO12, JO26, JO35), isolated from the intestine of fish and shrimp, on mucosal injury caused by *Aeromonas hydrophila* in crucian carp under an oral challenge model was investigated. The result showed that compared with LGG, all three *L. sakei* strains alleviated *A. hydrophila* induced intestinal barrier damage and inflammation (downregulated intestinal TNF-*α*/IL-1β, upregulated IL-10, and reduced MyD88) in crucian carp. *L. sakei* JO35 delivered the greatest improvement in growth and feed efficiency. Compared with the model group, *L. sakei* JO26 and JO35 significantly decreased the levels of serum acid phosphatase (ACP) and increased intestinal lysozyme, whereas *L. sakei* JO12 lowerd serum ACP but exacerbated the elevation of intestinal AKP. Microbiome and transcriptome analysis revealed that the protective effect of *L. sakei* may be associated with the strain’s intestinal colonization capacity and its regulation of phagolysosomal competence (lysosome/phagosome, LAMP) and IgA barrier via pIgR (prominent with JO35).

## 1 Introduction

With the rapid growth of the global population and the escalating demand for food security, aquaculture has become a vital component in securing sustainable protein sources for humanity. According to data from the United Nations Food and Agriculture Organization (FAO), global aquatic product output reached 179 mill54ion tons in 2022, with farmed aquaculture accounting for over 55% of this total for the first time—a landmark transition from reliance on wild fisheries toward the sustainable cultivation of aquatic resources (1). Aquatic products are characterized by high protein content, low fat levels, and a rich profile of essential amino acids and trace elements, serving as a primary dietary protein source for approximately 3 billion people worldwide, a role that is especially critical in developing countries (2). Furthermore, these products are abundant in micronutrients such as calcium, iron, and vitamin A, which are essential for preventing deficiency-related diseases (3). Notably, the presence of long-chain omega-3 fatty acids, including docosahexaenoic acid (DHA) and eicosapentaenoic acid (EPA), contributes to cardiovascular health and supports neurodevelopment and visual function (4). According to the 2022 China Fisheries Yearbook, China’s total aquatic product output had reached 68.659 million tons, with aquaculture contributing 55.655 million tons, representing 81.06% of the total (5). Jiangsu Province, a leading aquaculture region in China, produced 1.243 million tons of aquatic products in 2023, consistently ranking among the top producers nationwide. The province’s distinctive aquaculture systems include ecologically optimized shrimp-crab polyculture, pond-based healthy freshwater fish farming, and integrated marine shrimp-crab-shellfish cultivation (6).

Despite the high yield and profitability of current aquaculture practices, this model has contributed to significant environmental degradation, including the accumulation of nutrients and organic matter in aquatic systems, which promotes the proliferation of pathogenic bacteria and frequent disease outbreaks among cultured species (7). Bacterial infections are a major cause of economic losses in aquaculture, severely undermining the sector’s profitability. Predominant bacterial pathogens in fish farming include species of *Vibrio*, *Aeromonas*, *Edwardsiella*, *Flavobacterium*, *Pseudomonas*, and *Micrococcus* (8). To mitigate these losses, antibiotics such as sulfonamides, tetracyclines, quinolones, and *β*-lactams are commonly administered to enhance survival rates in aquatic animals (9). However, the widespread and often inappropriate use of antibiotics has driven the emergence of antibiotic-resistant bacteria within aquaculture environments (10). These resistant bacteria and associated resistance genes pose a significant public health risk, as they can be transmitted to humans via the food chain or through direct contact (11). Furthermore, antibiotic misuse disrupts the gut microbiota of aquatic animals, leading to dysbiosis characterized by diarrhea, bloating, intestinal inflammation, and impaired immune function (12).

.Recent studies have demonstrated that targeted probiotic supplementation across diverse aquaculture species enhances growth performance, optimizes gut microbiota composition, strengthens innate and adaptive immune functions, and improves disease resistance, underscoring probiotics as effective antibiotic alternatives in intensive systems (13). For instance, dietary supplementation with *Bacillus subtilis* W2Z enhanced crayfish resistance to *Aeromonas hydrophila* by increasing activities of immune-related enzymes and improving intestinal microbiota composition (14). At a dietary concentration of 10^8^ CFU/g, *Clostridium butyricum* significantly improved growth performance, beneficially reshaped the gut microbiota, modulated immune markers (downregulating IL-1β, IL-8, NF-κB, MyD88, and TNF-*α* while increasing serum lysozyme and IgM), and increased resistance to *A. salmonicida* in triploid rainbow trout (15). Zhang et al. isolated four *Bacillus* strains with broad-spectrum antibacterial activity from healthy crucian carp; these strains enhanced innate intestinal immunity by reducing pathogenic colonization (16). Similarly, Tan et al. isolated a *Staphylococcus* sp. from Nile tilapia and demonstrated that diets containing this strain improved growth, immunity, disease resistance, and overall intestinal health (17). Moreover, diets supplemented with *Lacticaseibacillus rhamnosus* GG (LGG) preserved intestinal barrier integrity in red tilapia, enhanced immune capacity, and improved growth and health status, supporting the direct inclusion of LGG in aquafeeds as a nutritional supplement (18).

*Latilactobacillus sakei,* belonging to the genus *Latilactobacillus,* was first described by Katagiri et al. (19). It is a lactic acid bacterium and was included in China’s list of edible microbial species in 2014, recognizing its safety and suitability for use in food and related applications. It is widely distributed in fermented foods, including flour (20), sourdough (21), fermented cabbage, sausage, sake, as well as in natural environments (22). Research has shown that it is one of the dominant bacterial species in meat products (23). *L. sakei* exhibits a range of probiotic functions, including inhibiting the growth of pathogenic bacteria, modulating the host immune system, enhancing intestinal barrier function, and improving gut microbiota balance. A growing body of research has demonstrated that *L. sakei* can produce a series of bacteriocins (24) and displays strong antagonistic activity against *Listeria monocytogenes* (25) and *Staphylococcus aureus* (26). For instance, *L. sakei* L115 exhibited inhibitory activity against *Listeria monocytogenes*, achieving a 31% reduction in the maximum specific growth rate and a 36% decrease in the maximum population density at 4 °C (27). Another study showed that *L. sakei* 1,018 in vacuum-packaged beef significantly inhibited the recovery of *Escherichia coli* throughout the entire storage period following high-pressure processing (28). Reyes-Becerril et al. reported that *L. sakei* 5–4 exerted a significant ameliorative effect on physiological and inflammatory markers in *Carassius auratus* infected with *A. hydrophila* (29). Further studies by the same group demonstrated that *L. sakei* supplementation improved the immune status and antioxidant capacity of *Lutjanus peru* (30). *L. sakei* MN1 was also reported to competitively inhibit the colonization of *Vibrio anguillarum* in the zebrafish gut by occupying adhesion sites (31).

Despite mounting evidence supporting probiotics as viable antibiotic alternatives in aquaculture, strain- and host-specific efficacy remains insufficiently characterized, particularly for *L. sakei* in freshwater cyprinids. Given *L. sakei*’s documented antimicrobial activity, immunomodulatory effects, and capacity to stabilize intestinal microbiota across food systems and selected marine and model fish species, its targeted application in crucian carp warrants systematic evaluation. In this study, we investigated the effects of dietary *L. sakei* strains (JO12, JO26, JO35), compared with LGG, on growth performance, non-specific immunity, resistance to *A. hydrophila*, gut microbial composition, and intestinal transcriptomic profiles in *Carassius auratus*.

## 2 Materials and methods

### 2.1 Bacterial species and culture conditions

*Aeromonas hydrophila* was cultured in Luria-Bertani (LB) medium at 28 °C. *L. sakei* strains JO12, JO26, and JO35 were isolated from the intestinal contents of fish and shrimp. LGG was used as the positive control strain. All Lactobacilli were cultured in MRS medium at 37 °C and preserved in glycerol tubes at −80 °C.

### 2.2 Antimicrobial assay

The *A. hydrophila* strain was streaked onto LB agar and incubated at 28 °C for 24 h to obtain single colonies. A single colony was then inoculated into LB broth to prepare a bacterial suspension, and the optical density was adjusted to OD_600 nm_ = 0.5 by spectrophotometry. Meanwhile, *L. sakei* strains JO12, JO26, JO35, and LGG were revived from −80 °C stocks and subcultured twice for activation. Second-passage cultures were incubated at 37 °C for 18 h, and the suspensions were standardized to 1 at OD_600 nm_ = 1.0. Sterile Petri dishes were prepared under a laminar flow hood by pouring 15–18 mL of LB broth agar and allowing it to solidify. Subsequently, 100 μL of the *A. hydrophila* suspension was evenly spread onto the agar surface. Sterilized Oxford cups were gently placed on the inoculated plates, and 200 μL of each Lactobacilli suspension in MRS broth (OD_600 nm_ = 1.0) was carefully pipetted into the corresponding Oxford cups. Plates were incubated upright at 37 °C for 18 h. Following incubation, the diameters of the inhibition zones around each cup were measured and recorded. All assays were performed in triplicate.

### 2.3 Animal models

**Figure 1:**
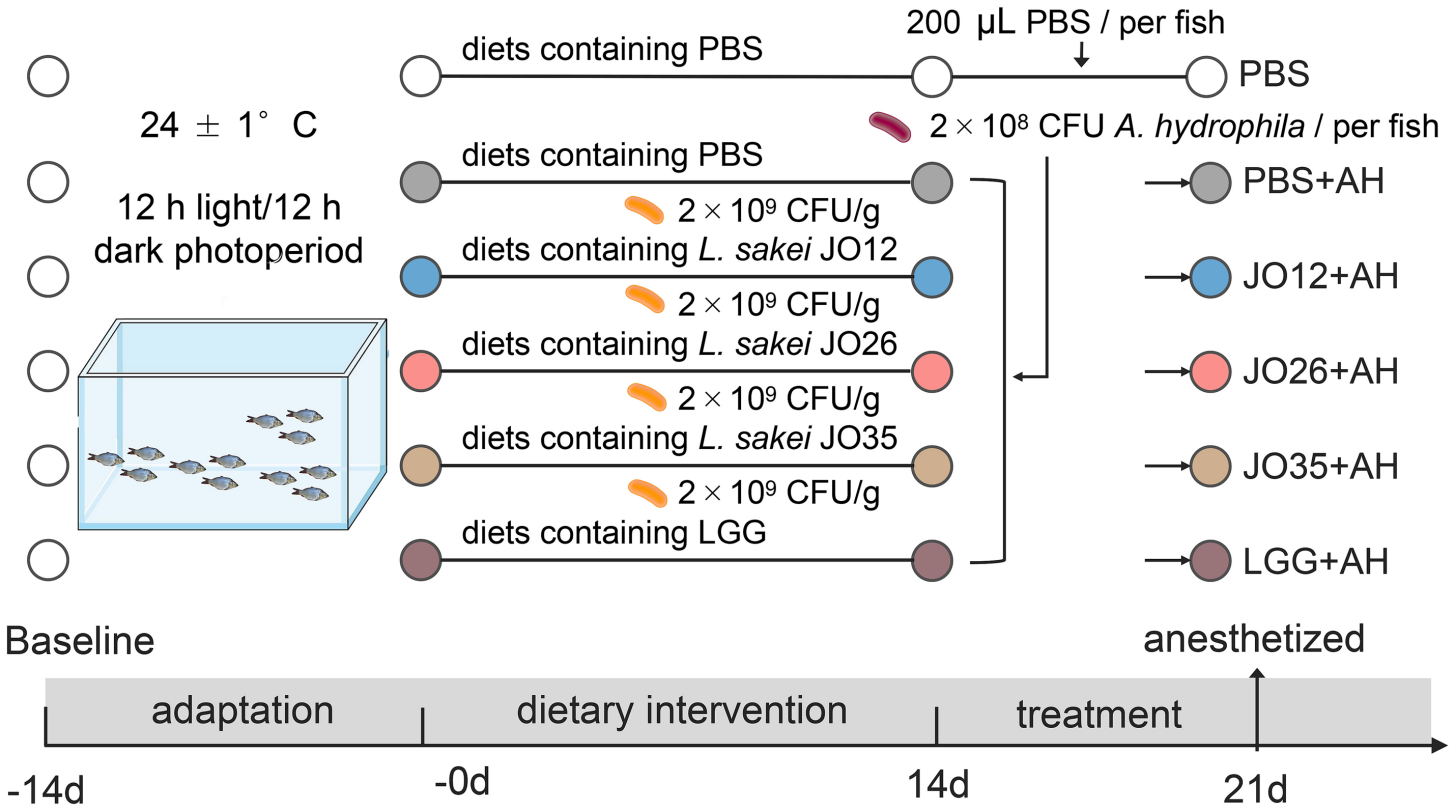
Design of animal experiment.

All procedures involving animals were approved by the Ethics Committee of Jiangsu Ocean University, China [NO: Jou2024032411 (3)]. A total of 120 crucian carp (*Carassius auratus*) with an average body length of 8.3 ± 0.3 cm were used. 120 fish were randomly assigned to six groups (PBS, PBS + AH, JO12 + AH, JO26 + AH, JO35 + AH, LGG + AH) and housed in six glass tanks (80 × 50 × 50 cm), each equipped with a closed recirculating aquaculture system (RAS) in glass tanks under laboratory conditions (24 ± 1 °C, 12 h light/12 h dark photoperiod) for 2 weeks. During the adaption period, all fish were fed a commercial diet at 2% of body weight, twice daily. Throughout the experiment, water quality parameters were monitored daily, maintaining pH 7.8 ± 0.5, ammonia nitrogen <0.5 mg/L, nitrite <0.05 mg/L, and dissolved oxygen at approximately 7.0 mg/L. After the adaptation period, fish in the six groups were fed diets containing PBS or *L. sakei* strains (2 × 10^9^ CFU/g) for 8 weeks. Then, each group was orally gavage of 200 μL sterile PBS or *A. hydrophila* (2 × 10^8^ CFU per fish) for seven consecutive days. The animal experimental design is shown in Figure 1 and the experimental procedures were performed as previously described (32).

### 2.4 Growth performance measurement

During the animal experiment, the following growth performance parameters were recorded for each group of crucian carp: initial average body weight (g), final average body weight (g), initial average body length (cm), final average body length (cm), weight gain rate (WGR, %), specific growth rate (SGR, %/d), feed converson ratio (FCR), and condition factor (CF, %). The indices were calculated as follows:

- Initial average body weight (g) = Initial total weight/Initial number of fish.
- Final average body weight (g) = Final total weight/Final number of fish.
- Initial average body length (cm) = Initial total length/Initial number of fish.
- Final average body length (cm) = Final total length/Final number of fish.
- Weight gain rate (WGR, %) = (Final total weight–Initial total weight)/Initial total weight × 100.
- Specific growth rate (SGR, %/d) = [ln (Final average body weight)–ln (Initial average body weight)]/Experimental days × 100.
- Feed conversion ratio (FCR) = Feed intake/(Final average body weight–Initial average body weight).
- Condition factor (CF, %) = (Final body weight / (Final body length)^3^) × 100.

### 2.5 Sample collection

After the infection experiment, all fish were anesthetized with 300 mg/L tricaine methane sulfonate (MS-222). Blood samples were collected from the caudal vein using disposable 1 mL syringes pre-moistened with heparin sodium solution (2,800 U/mL). The collected blood was kept in the dark at room temperature for 1 h and then centrifuged at 3,000 rpm for 15 min. Serum was transferred to 2 mL microcentrifuge tubes and stored at −20 °C for subsequent biochemical analyses. Following dissection, three fish per group were selected. A 1 cm segment of mid-intestine was excised and fixed in 4% paraformaldehyde for histology. The remaining intestinal tissues were placed in 2 mL cryovials, snap-frozen in liquid nitrogen, and stored at −80 °C for non-specific immune assays and transcriptomic analysis. Intestinal contents were collected by gentle extrusion, transferred to 2 mL cryovials, rapidly frozen in liquid nitrogen, and stored at −80 °C for gut microbiota diversity profiling and sequencing.

### 2.6 Histological assessment

Mid-intestinal tissue samples (approximately 5 mm) were fixed in 4% neutral-buffered paraformaldehyde, dehydrated, and embedded in paraffin. Sections 4 μm thick were prepared and stained with hematoxylin and eosin (H&E). The stained sections were air-dried for 2 weeks and then examined under a light microscope. Tissue morphology was evaluated at multiple magnifications (33).

### 2.7 Analysis of biochemical indicators

To assess biochemical parameters in the serum and intestinal tissues of *A. hydrophila*–infected crucian carp fed with *L. sakei*, approximately 1 cm of intestinal tissue was homogenized in 1 mL of RIPA lysis buffer. The homogenate was centrifuged at 14,000 × g for 15 min at 4 °C, and the supernatant was collected. Total protein concentration was determined using a protein assay kit (Shanghai Yuanye Bio, China). The activities of alkaline phosphatase (AKP), acid phosphatase (ACP), and alanine aminotransferase (ALT) were measured with the corresponding enzyme assay kits (Nanjing Jiancheng Bioengineering, China). Lysozyme (LZM) activity was quantified using a lysozyme assay kit (Shanghai Meilian Bio, China). The activities of LZM, ACP, AKP, and AKP in intestinal tissues were normalized to protein content and expressed as units per gram of protein (U/gprot). Serum LZM, ACP, AKP, and ALT activities were expressed as units per milliliter of serum (U/mL).

### 2.8 Cytokine assay

Enzyme-linked immunosorbent assay (ELISA) kits (Shanghai Keqiao Bio, China) specifically developed and validated for fish were used to quantify tumor necrosis factor-*α* (TNF-α), interferon-*γ* (IFN-γ), interleukin-10 (IL-10), interleukin-1β (IL-1β), and myeloid differentiation primary response 88 (MyD88) in serum and intestinal tissues. The ELISA kits were fish TNF-α ELISA kit (KQ114000), fish IL-1β ELISA kit (KQ139942), fish IL-10 ELISA kit (KQ109400), fish IFN-γ ELISA kit (KQ109401), and fish MyD88 ELISA kit (KQ124877). The ELISAs employed a double-antibody sandwich format. Microplates were pre-coated with purified capture antibodies. Samples were added to the wells, followed by horseradish peroxidase (HRP)–conjugated detection antibodies, forming an antibody–antigen–HRP complex. After thorough washing, tetramethylbenzidine (TMB) substrate was added for color development. TMB was catalyzed by HRP to produce a blue product that turned yellow upon acidification. Color intensity was directly proportional to the cytokine concentration in the sample. Absorbance was measured at 450 nm using a microplate reader, and cytokine concentrations were calculated from standard curves.

### 2.9 Determination of intestinal flora

A 0.05 g aliquot of each intestinal content sample was mixed with magnetic beads and homogenized using a tissue disruptor. Bacterial DNA was extracted, and the V3–V4 region of the 16S rRNA gene was amplified and purified. A sequencing library was then constructed from the amplicons, and paired-end sequencing was performed on an Illumina platform. Raw reads underwent quality control, including merging and filtering, followed by operational taxonomic unit (OTU) clustering or amplicon sequence variant (ASV) denoising. Alpha- and beta-diversity analyses were performed using QIIME 2 (34).

### 2.10 Transcriptome analysis

Total RNA was extracted from a 0.05 g portion of each intestinal tissue sample, and mRNA was enriched. The mRNA was fragmented, first-strand cDNA was synthesized using random hexamer primers, and second-strand cDNA was subsequently synthesized. After end repair, A-tailing, adapter ligation, fragment selection, amplification, and purification, the sequencing library was prepared. The library was quantified, and fragment size distribution was assessed. Libraries that passed quality control were pooled according to effective concentration and target sequencing depth, and sequenced on an Illumina platform. Raw reads were processed with fastp to obtain high-quality clean reads. Clean data quality metrics (Q20, Q30) and GC content were evaluated. The reference genome index was built using HISAT2 (v2.0.5), and paired-end clean reads were aligned to the reference genome with HISAT2 (v2.0.5). Novel gene prediction was performed using StringTie (v1.3.3b). Differentially expressed genes (DEGs) were analyzed for Gene Ontology (GO) enrichment using clusterProfiler (v3.8.1), and Kyoto Encyclopedia of Genes and Genomes (KEGG) pathway enrichment analysis was conducted to identify functionally significant pathways (35).

### 2.11 Statistical analysis

Statistical analyses and graphical representations were performed using GraphPad Prism (v8.02; San Diego, CA, United States). Data are expressed as mean ± standard error of the mean (SEM). The Shapiro–Wilk test was used to assess normality, and Levene’s test was used to evaluate homogeneity of variances. One-way analysis of variance (ANOVA), followed by *post hoc* pairwise comparisons, was conducted to determine significant differences between groups.

## 3 Results

### 3.1 Antagonistic activity of *Latilactobacillus sakei* against *Aeromonas hydrophila*

Based on our prior experiments, 30 *L. sakei* strains (JO12, JO16, JO21, JO26, JO29, JO34, JO35, JO41, JO48, JO61, JO62, JO63, JO64, JO94, JO95, JO97, JO101, JO102, JO104, JO105, JO106, JO113, JO114, JO115, JO117, JO118, JO123, JO141, JO142 and JO312) isolated from the intestinal contents of fish and shrimp were initially screened for *in vitro* antagonistic activity against *A. hydrophila*. Among these, three *L. sakei* strains (JO12, JO26 and JO35) with the strongest antagonistic activity against *A. hydrophila*, along with LGG, were selected for evaluation. Antagonistic activity was assessed using the Oxford cup assay, and the results are presented in Table 1. All strains exhibited measurable inhibitory effects. The Inhibition zones of the three *L. sakei* strains were greater than 15 mm, with no significant differences among them. The antibacterial ability of all three *L. sakei* strains was higher than that of LGG.

**Table 1:** Antagonistic activity of three *L. sakei* strains and LGG against *Aeromonas hydrophila.*

Groups	JO12	JO26	JO35	LGG
Zone of inhibition(mm)	16.13 ± 0.35^a^	15.07 ± 0.98^a^	15.20 ± 0.66^a^	12.03 ± 0.49^b^

### 3.2 Effects of *Latilactobacillus sakei* on the growth performance of crucian carp

The growth parameters of crucian carp in all groups are presented in Table 2. All probiotic treatments improved the weight gain rate (WGR) of crucian carp relative to the PBS and PBS + AH controls. *L. sakei* JO35 significantly increased both WGR and specific growth rate (SGR), outperforming all other groups. *L. sakei* JO12 and *L. sakei* JO26 also significantly enhanced WGR and SGR compared with the PBS and PBS + AH treatments. Crucian carp treated with *L. sakei* JO35 exhibited the lowest feed conversion ratio (FCR), and *L. sakei* JO12 and *L. sakei* JO26 likewise reduced FCR relative to PBS and PBS + AH. The condition factor (CF) did not differ significantly among groups.

**Table 2:** Effect of *L. sakei* on the growth performance of crucian carp.

Groups	Growth parameter					
	Initial weight (g)	Final weight (g)	WGR (%)	SGR (%)	FCR	CF (%)
JO12 + AH	14.60 ± 1.10^a^	19.42 ± 2.29^b^	33.01 ± 1.19^b^	0.67 ± 0.10^ab^	2.18^e^	2.22 ± 0.17^a^
JO26 + AH	15.01 ± 0.93^a^	19.48 ± 2.87^b^	31.38 ± 1.95^bc^	0.64 ± 0.20^ab^	2.23^d^	2.41 ± 0.22^a^
JO35 + AH	14.23 ± 0.86^a^	20.47 ± 3.31^b^	43.85 ± 2.45^a^	0.85 ± 0.24^a^	1.68^f^	2.47 ± 0.27^a^
LGG + AH	14.60 ± 0.89^a^	18.84 ± 3.12^ab^	29.04 ± 2.23^c^	0.59 ± 0.25^ab^	2.48^c^	2.17 ± 0.25^a^
PBS + AH	15.80 ± 0.95^a^	18.64 ± 3.22^ab^	17.97 ± 2.27^d^	0.37 ± 0.27^b^	3.70^b^	2.09 ± 0.33^a^
PBS	14.35 ± 0.88^a^	16.90 ± 1.83^a^	17.77 ± 0.95^d^	0.38 ± 0.11^b^	4.12^a^	2.11 ± 0.23^a^

Data in the same column with the same superscript without letters or lowercase letters indicate non-significant differences (*p* > 0.05), while different superscripts with lowercase letters indicate significant differences (*p* < 0.05). Data are presented as mean ± standard deviation (means ± SD, *n* = 3), and letters represent the level of significant difference for each group, *p* < 0.05.

### 3.3 Effect of *Latilactobacillus sakei* on the intestinal histological structure of crucian carp

**Figure 2:**
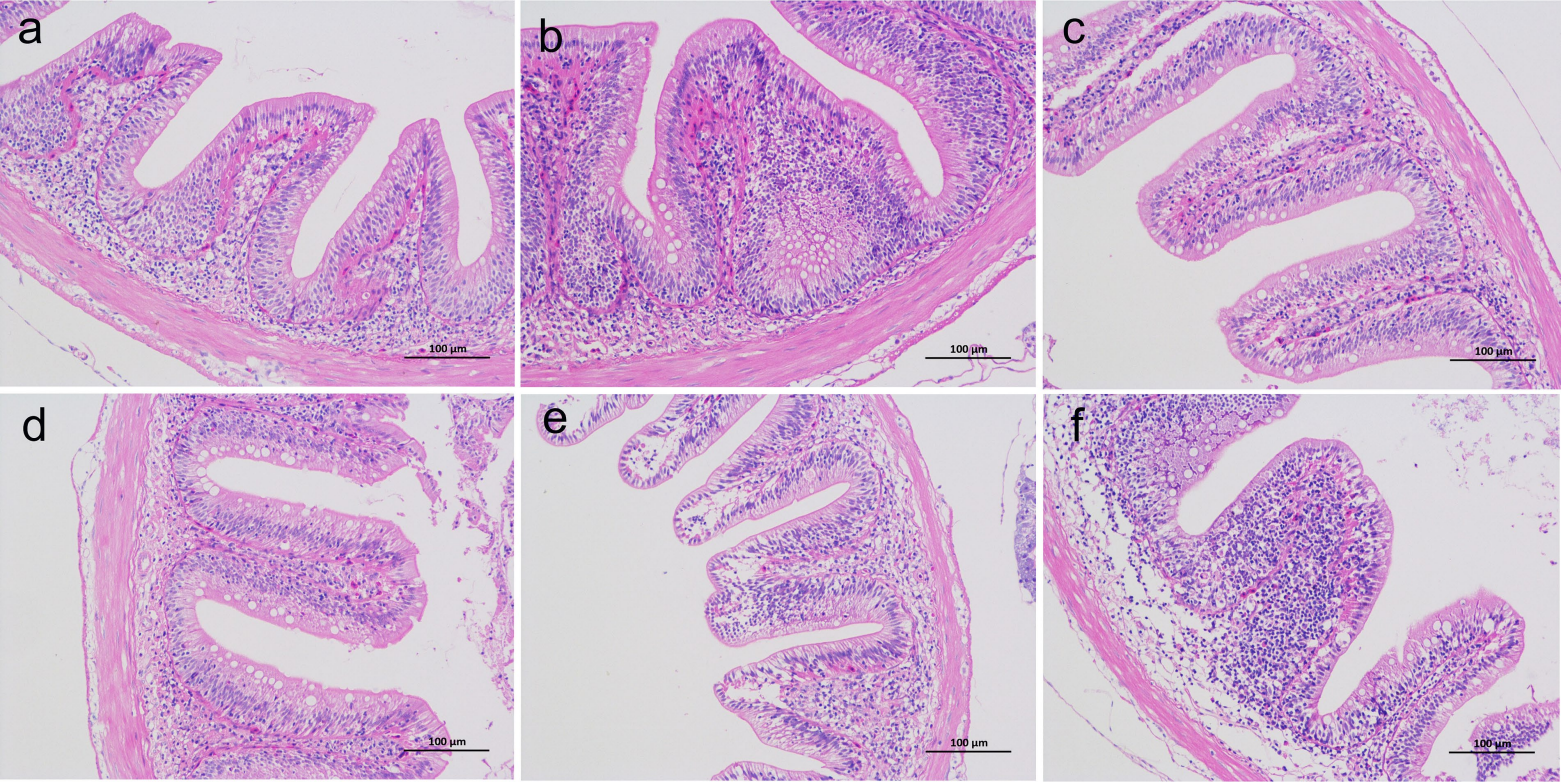
Effect of *L. sakei* on the intestinal histological structure of Crucian carp **(a–f)**: PBS group **(a)**, PBS + AH group **(b)**, JO12 + AH group **(c)**, JO26 + AH group **(d)**, JO35 + AH group **(e)**, and LGG + AH group **(f)**.

As shown in Figure 2, the layered architecture of the intestinal tissue in the PBS group was clear and intact, with an undamaged mucosal epithelium. Goblet cells were distinctly distributed, and no inflammatory cell infiltration was observed in the submucosa. In the PBS + AH group, mucosal edema was evident, accompanied by inflammatory cell infiltration in the mucosa and submucosa. Treatment with *L. sakei* JO12, JO26, and JO35 alleviated mucosal edema and reduced lymphocytic infiltration in the intestines of crucian carp. By contrast, the intestinal tissue of fish treated with LGG showed fewer goblet cells, mucosal swelling, epithelial injury, and more pronounced inflammatory cell infiltration compared with the PBS group.

### 3.4 Effect of *Latilactobacillus sakei* on biochemical indexes in the serum and intestine of crucian carp

**Figure 3:**
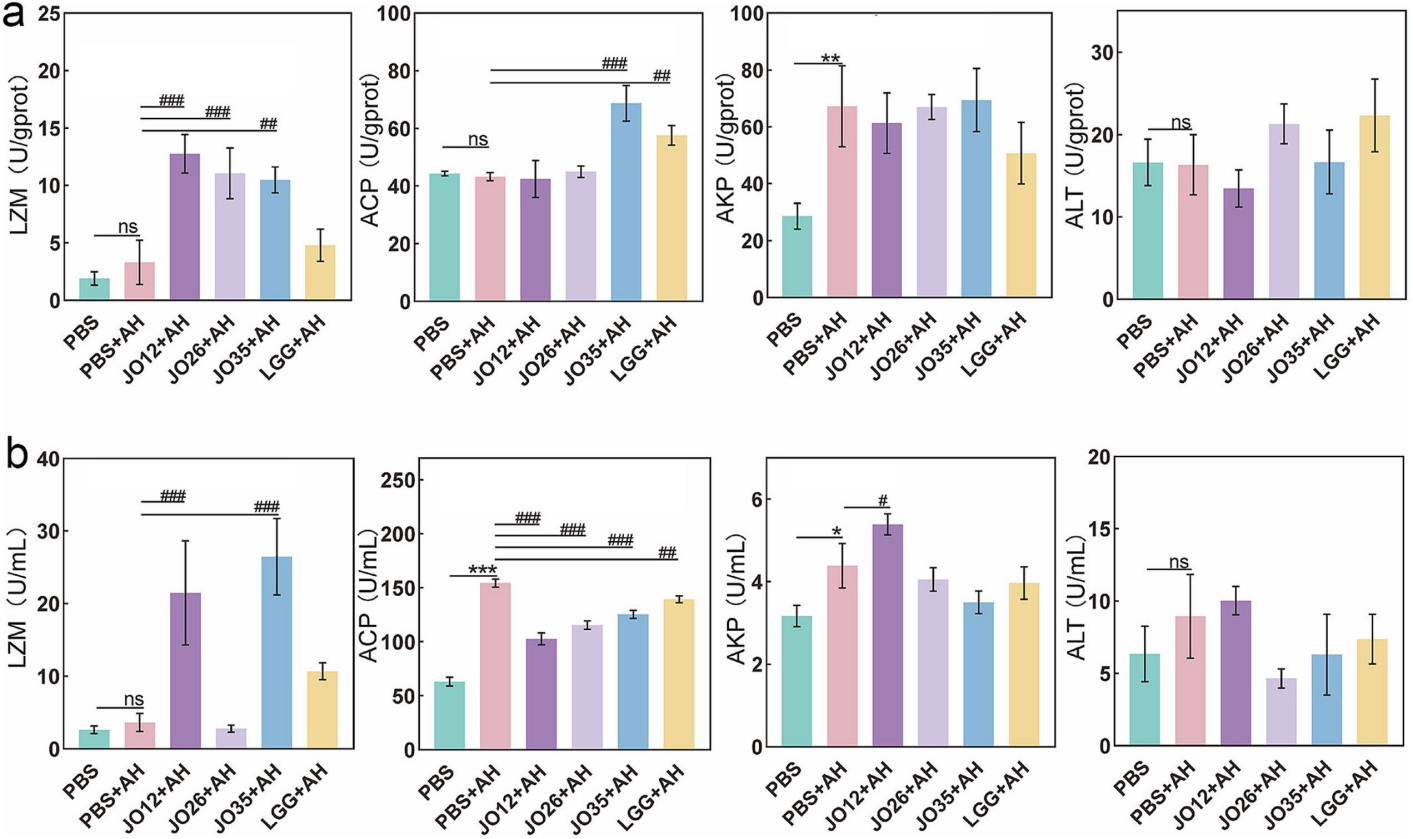
**(a)** Biochemical indexes in intestinal tissues (enzyme activities were normalized to protein content and expressed as U/g prot); **(b)** biochemical indexes in serum of crucian carp (enzyme activities were expressed as U/mL); LZM, lysozyme; ACP, acid phosphatase; AKP, alkaline phosphatase; ALT, alglutamyltransferase.

The effects of *L. sakei* on lysozyme, acid phosphatase (ACP), alkaline phosphatase (AKP), and alanine aminotransferase (ALT) in the serum and intestines of crucian carp infected with *A. hydrophila* were evaluated, as shown in Figure 3. Compared with the PBS group, *A. hydrophila* infection caused a significant increase in intestinal AKP, whereas intestinal lysozyme, ACP, and ALT did not change markedly. Relative to the PBS + AH group, *L. sakei* JO35 significantly increased serum lysozyme and ACP, while *L. sakei* JO12 and JO26 significantly increased serum lysozyme. In the serum, *A. hydrophila* infection significantly increased ACP and AKP levels and did not alter lysozyme or ALT. Treatment with *L. sakei* JO12, JO26, JO35, and LGG reduced serum ACP in *A. hydrophila*–infected fish. Moreover, *L. sakei* JO12 and JO35 significantly increased serum lysozyme levels in infected crucian carp (Figure 3).

### 3.5 Effect of *Latilactobacillus sakei* on the expression of intestinal inflammatory factors in crucian carp

**Figure 4:**
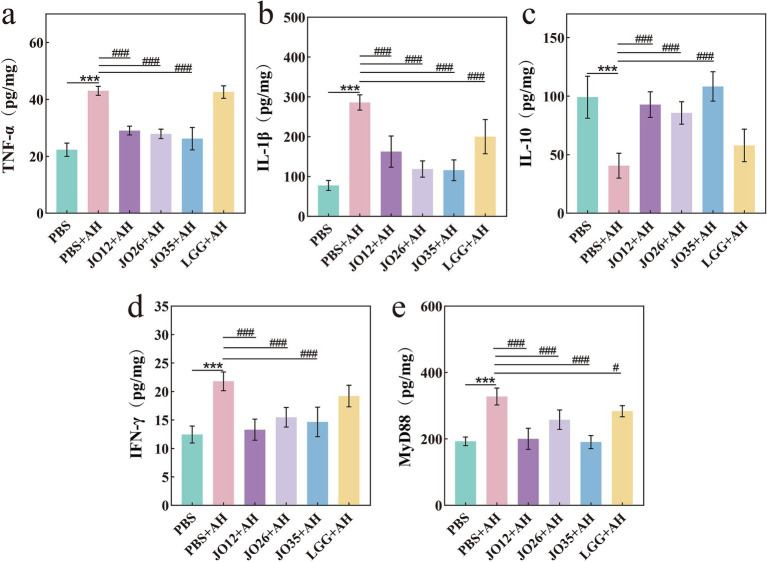
Effect of *L. sakei* on the expression of intestinal inflammatory factors of crucian carp **(a–e)**.

The levels of interferon-gamma (IFN-*γ*), interleukin-1β (IL-1β), interleukin-10 (IL-10), myeloid differentiation primary response 88 (MyD88), and tumor necrosis factor-AKPha (TNF-*α*) in intestinal tissue were quantified. As shown in Figure 4, *A. hydrophila* infection (PBS + AH) elicited a robust pro-inflammatory response, evidenced by significant increases in TNF-α, IL-1β and MyD88 relative to PBS controls, along with decreases in IL-10 and IFN-γ. Supplementation with *L. sakei* markedly attenuated this pro-inflammatory surge. All tested *L. sakei* strains reduced TNF-α, IL-1β, and IFN-γ compared with the PBS + AH group. Concomitantly, *L. sakei* JO12, JO26, and JO35 increased the anti-inflammatory cytokine IL-10 in the intestines of infected fish, indicating that probiotic intervention alleviated *A. hydrophila*–induced intestinal inflammation. MyD88, a key adaptor in TLR signaling, was significantly upregulated by infection but was downregulated by *L. sakei* JO12, JO26, and JO35, suggesting mitigation of TLR–MyD88–dependent inflammatory signaling.

### 3.6 Effect of *Latilactobacillus sakei* on the intestinal flora of crucian carp

**Figure 5:**
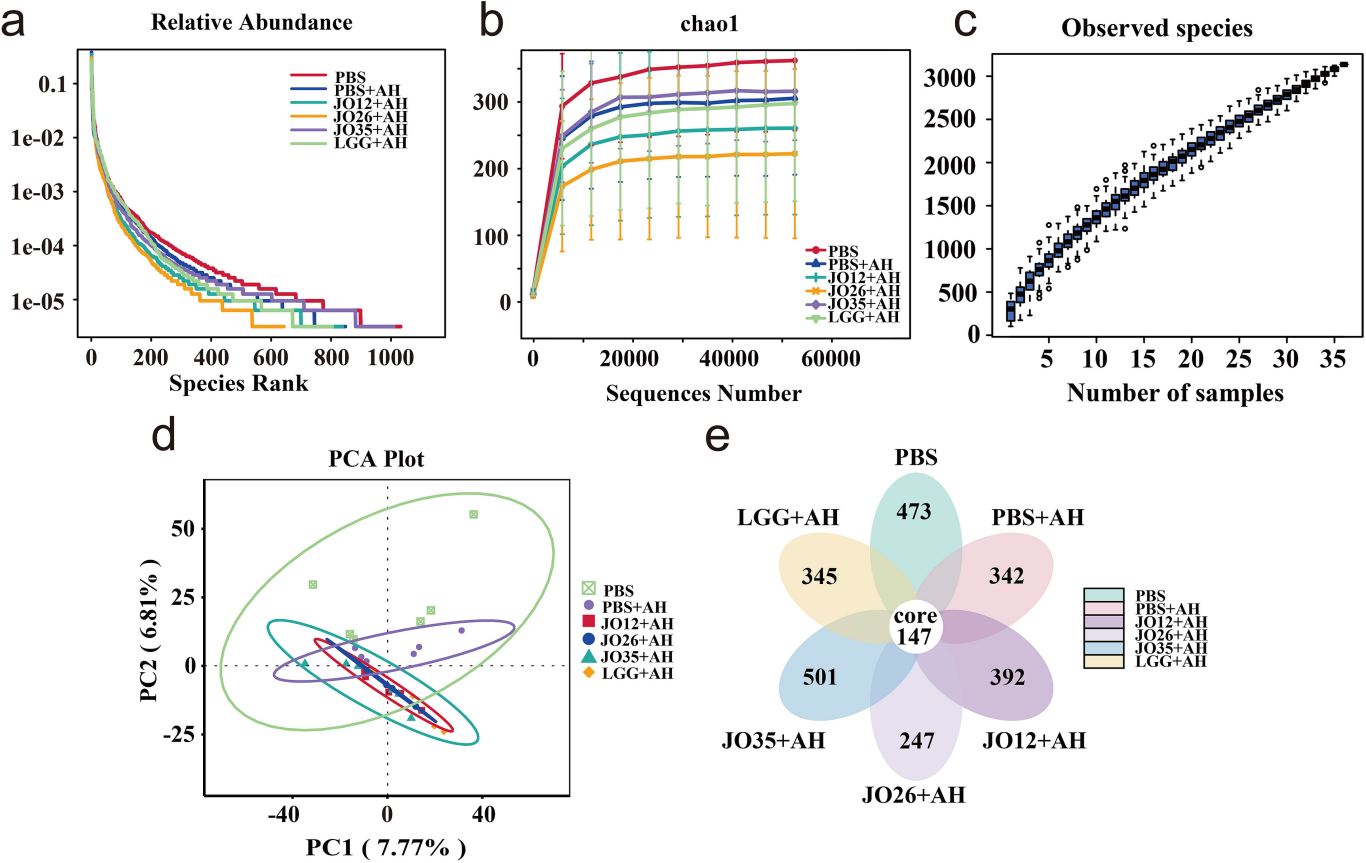
Sequencing depth and diversity analysis based on OTUs. **(a)** Rarefaction curve, the horizontal coordinate is the number of sequencing strips randomly selected from the samples, the vertical coordinate is the number of OTUs obtained based on the number of sequencing strips, and different samples are represented by using different colored curves; **(b)** Chao1 curve, the horizontal coordinate is the ordinal number sorted by the abundance of OTUs, the vertical coordinate is the relative abundance of the corresponding OTUs, different samples are represented using different colored dash lines; **(c)** Species accumulation curve; **(d)** PCA plot; **(e)** Vene diagram.

As shown in Figure 5, rarefaction curves plateaued for all groups, and species accumulation increased smoothly without sharp rises, indicating adequate sequencing depth and sample size for robust diversity assessment. The PBS group displayed the widest, flattest Chao1 curve, reflecting greater richness and evenness. Infection with *A. hydrophila* (PBS + AH) reduced both metrics and shifted community structure, as evidenced by clear separation from PBS in PCA space. A Venn diagram revealed 147 core features shared across groups. The number of unique features was highest in JO35 + AH and PBS, intermediate in JO12 + AH and PBS + AH, and lowest in JO26 + AH and LGG + AH, supporting a diversity-preserving effect most pronounced for *L. sakei* JO35.

**Figure 6:**
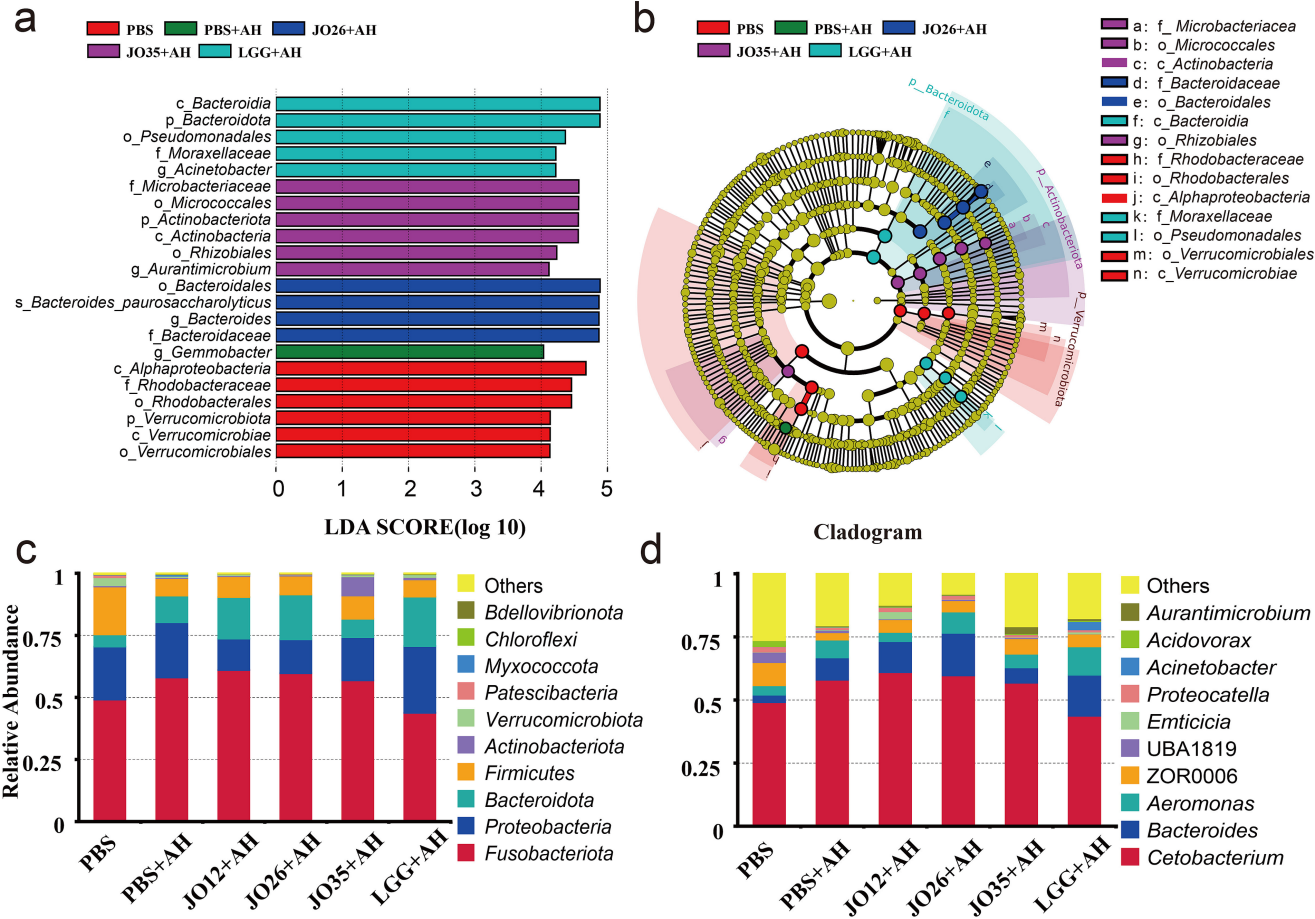
Effect of *L. sakei* on the intestinal flora of crucian carp. **(a)** Linear discriminant analysis (LDA) bar plot; **(b)** Column diagram of microbial composition (LEFSE analysis); **(c)** Diagram of the composition of the overall flora structure based on the phylum level; **(d)** Diagram of the composition of the overall flora structure based on the genus level.

As shown in Figure 6, linear discriminant analysis (LDA) bar plots and column charts indicate 21 dominant OTUs across the six groups. At the phylum level, the gut microbiota was dominated by Fusobacteriota, Proteobacteria, Bacteroidota, and Firmicutes across groups. Infection with *A. hydrophila* increased Proteobacteria, whereas *L. sakei* JO12 (12.69%), JO26 (13.67%), and JO35 (17.41%) reduced Proteobacteria levels in the intestines of crucian carp. Compared with the PBS group, *A. hydrophila* infection increased the relative abundance of Bacteroidota, whereas supplementation with *L. sakei* JO35 reduced Bacteroidota levels. Firmicutes decreased markedly in the intestines of crucian carp after *A. hydrophila* infection across all treatment groups compared with PBS. At the genus level, *Cetobacterium* constituted more than 40% of the community in all groups, with the highest relative abundance in JO12 + AH (61.01%) and the lowest in LGG + AH (43.75%). In the PBS group, *Bacteroides* accounted for 2.99%, whereas the proportions in PBS + AH, JO12 + AH, JO26 + AH, JO35 + AH, and LGG + AH were 8.84, 12.31, 16.89, 6.08, and 16.27%, respectively. The abundance of *Aeromonas*, a key pathogen indicator, was reduced most effectively by *L. sakei* JO12 (3.64%) and JO35 (5.42%) compared with PBS + AH (7.06%), but was higher in JO26 + AH (8.43%) and LGG + AH (11.20%).

**Figure 7:**
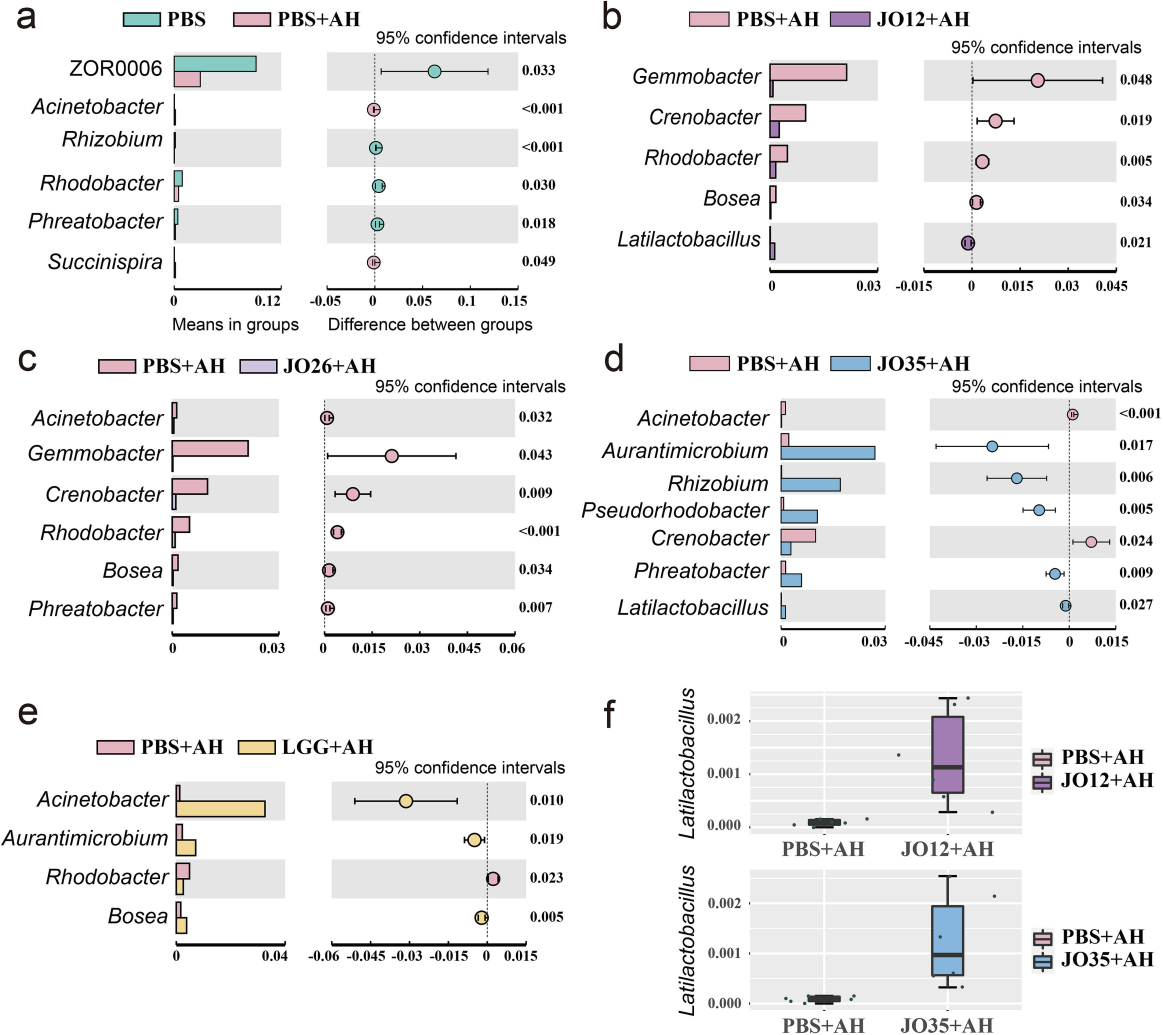
T-text analysis of the intestinal flora of crucian carp based on genus level. **(a)** PBS vs. PBS + AH; **(b)** PBS + AH vs. JO12 + AH; **(c)** PBS + AH vs. JO26 + AH; **(d)** PBS + AH vs. JO35 + AH; **(e)** PBS + AH vs. LGG + AH; **(f)** Abundance of *Latilactobacillus* in JO12 + AH and JO35 + AH group.

T-test analysis of the intestinal flora at the genus level showed that the PBS group had higher abundances of ZOR0006 and several putatively beneficial or environmental genera (e.g., *Rhizobium*, *Rhodobacter*, *Phreatobacter*), whereas the PBS + AH group was enriched in *Acinetobacter* and *Succinatimonas* (originally “Succinisipra”). Compared with the PBS + AH group, *L. sakei* JO12 significantly increased the abundance of *Latilactobacillus* in the intestine of crucian carp. *L. sakei* JO35 markedly increased the abundances of *Aurantimicrobium*, *Rhizobium*, *Pseudorhodobacter*, *Phreatobacter* (corrected from “Phrestobacter”), and *Latilactobacillus*. All three strains—*L. sakei* JO12, JO26, and JO35—attenuated the *A. hydrophila*–induced increase in *Acinetobacter*. LGG significantly increased the abundance of *Rhodobacter*. *Latilactobacillus* abundance was significantly higher in the JO12 + AH and JO35 + AH groups than in PBS + AH, with non-overlapping 95% confidence intervals and *p*-values below 0.05 (Figure 7).

### 3.7 Effect of *Latilactobacillus sakei* on the expression of transcriptional genes in the intestinal contents of crucian carp

**Figure 8:**
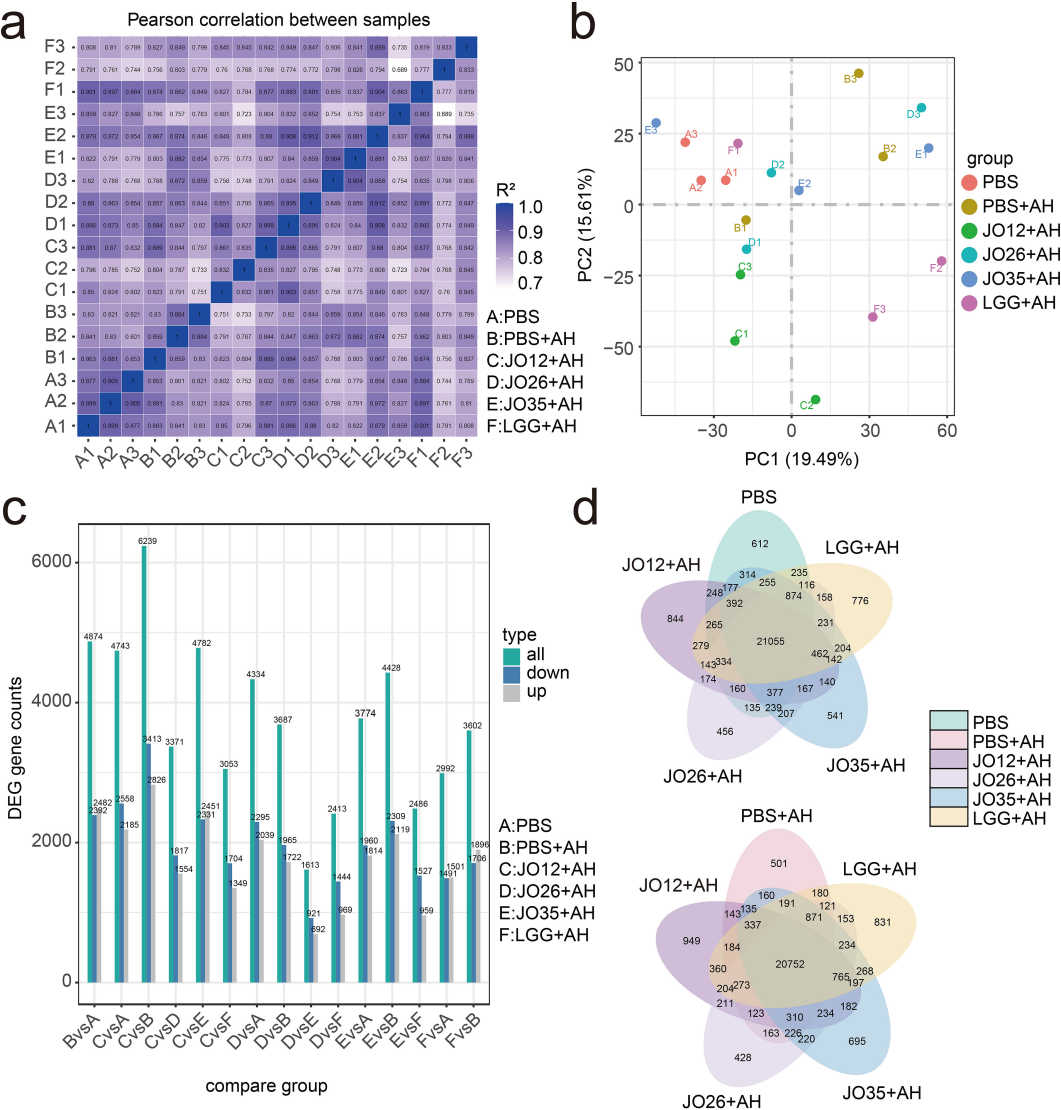
Effect of *L. sakei* on the transcriptional gene expression in the intestinal contents of crucian carp. **(a)** Heatmap of inter-sample peaeson correlation; **(b)** PCA plot of global gene expression; **(c)** Histogram of differentially expressed gene counts per pairwise comparison (up- and downregulated genes); **(d)** Vene diagrams of shared and unique differentially expressed genes among groups.

In Figure 8A, biological replicates clustered tightly with Pearson R^2^ values predominantly ≥0.95 within each treatment, confirming robust sequencing quality, whereas between-group correlations were lower, consistent with treatment-specific transcriptional programs. In Figure 8B, samples in the PBS + AH group separated strongly from those in the PBS group, indicating that *A. hydrophila* infection induced profound alterations in the intestinal transcriptomic landscape of crucian carp. Samples in the JO12 + AH and JO35 + AH groups shifted away from PBS + AH and toward PBS, while samples in the JO26 + AH and LGG + AH groups occupied intermediate positions, suggesting partial remediation. Consistent with these results, Figure 8c showed that *A. hydrophila* infection (B vs. A) yielded the largest number of differentially expressed genes (DEGs). Relative to PBS + AH, JO12 + AH and JO35 + AH displayed higher total DEG counts (with balanced up- and down-regulation), reflecting broader host regulatory effects, whereas JO26 + AH and LGG + AH exhibited fewer changes, indicative of weaker modulation. Figure 8d demonstrated a substantial core of infection-responsive genes shared among all infected groups. Notably, JO12 + AH and JO35 + AH possessed larger pools of unique DEGs and greater overlap with samples in the PBS group than with PBS + AH. Collectively, *L. sakei* JO12 and JO35 exerted the most pronounced corrective effects on *A. hydrophila*–induced dysregulation of the intestinal transcriptome in crucian carp, followed by LGG and *L. sakei* JO26.

**Figure 9:**
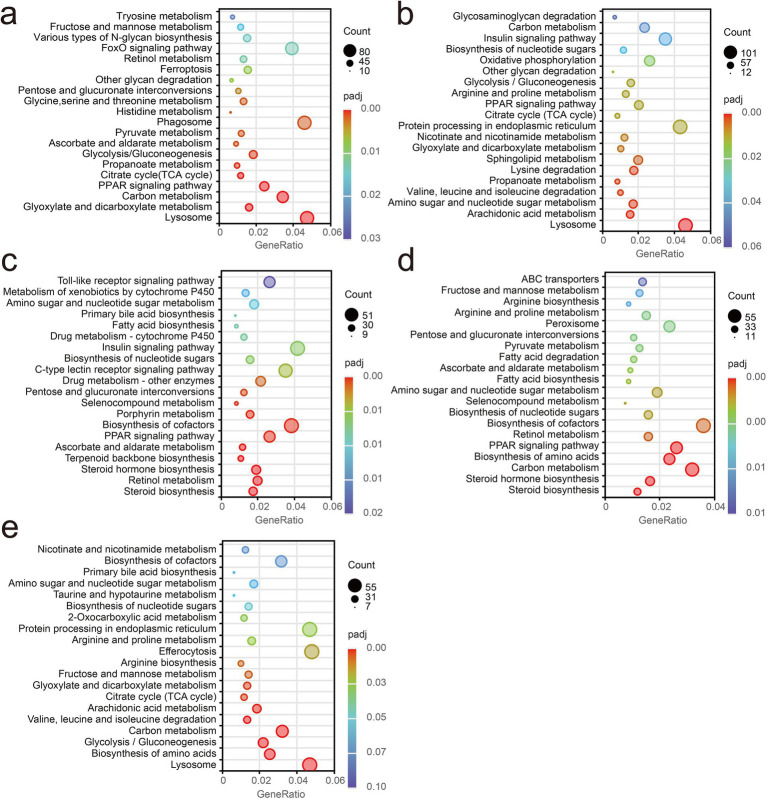
Bubble chart of top 20 significantly enriched KEGG pathways for each pairwise comparison: **(a)** PBS vs. PBS + AH; **(b)** PBS + AH vs. JO12 + AH; **(c)** PBS + AH vs. JO26 + AH; **(d)** PBS + AH vs. JO35 + AH; **(e)** PBS + AH vs. LGG + AH.

**Figure 10:**
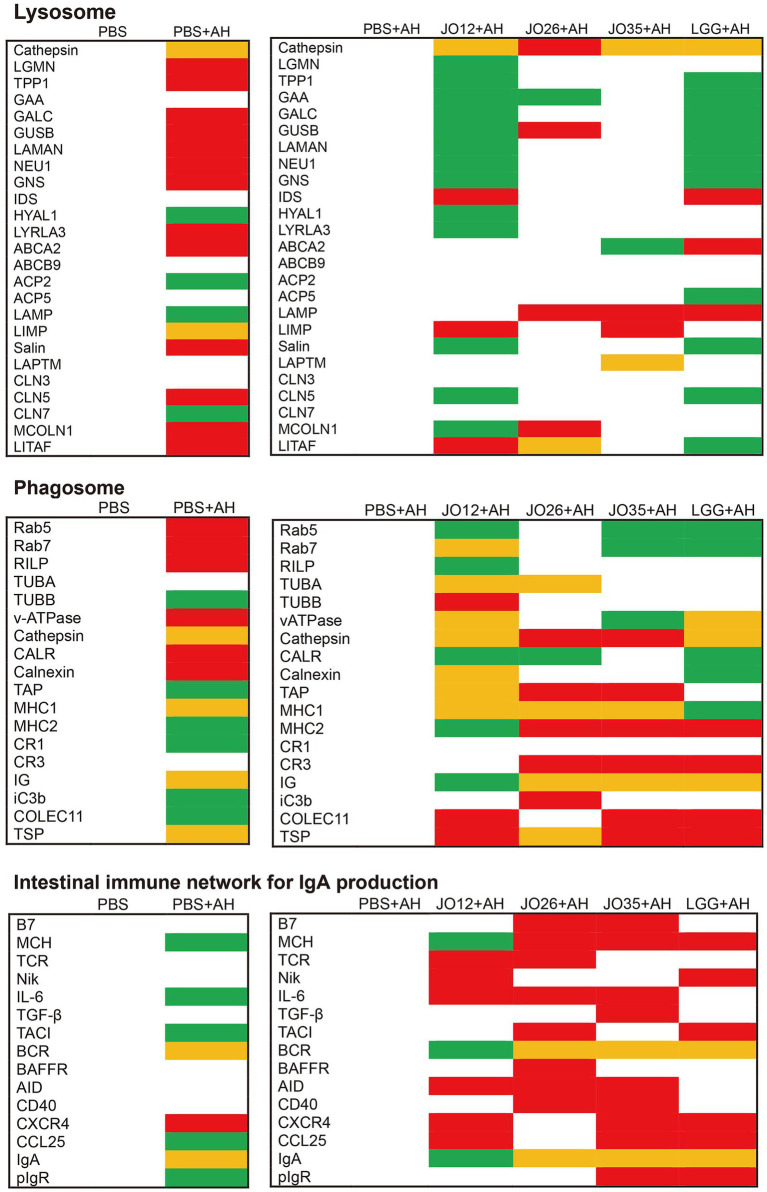
Differentially expressed genes enriched in key immune and metabolic pathways. Green indicating consistent downregulation in all three samples, red indicating consistent upregulation in all three samples, and yellow indicating inconsistent changes across samples.

The top 20 significantly enriched KEGG pathways for each pairwise comparison are shown in Figure 9. In Figure 9A, *A. hydrophila* infection elicited pronounced enrichment of innate immune and inflammatory pathways concomitant with metabolic reprogramming in crucian carp, including lysosome, phagosome, PPAR signaling, FoxO signaling, glycolysis/gluconeogenesis, the tricarboxylic acid (TCA) cycle, multiple amino acid and carbohydrate metabolic pathways, as well as retinol metabolism and ferroptosis-related processes. Relative to PBS + AH group, Figure 8B showed that *L. sakei* JO12 supplementation significantly enriched pathways in PPAR signaling, lysosome, glycosaminoglycan degradation, carbon metabolism and oxidative phosphorylation. *L. sakei* JO26 supplementation significantly enriched Toll-like receptor signaling, C-type lectin receptor signaling, PPAR signaling, amino acid and carbohydrate metabolic and drug metabolism (cytochrome P450). *L. sakei* JO35 supplementation significantly enriched peroxisome, PPAR signaling and multiple amino acid metabolic pathways. LGG supplementation significantly enriched efferocytosis, lysosome and nicotinate and nicotinamide metabolism (Figure 10).

Differentially expressed genes involved in the lysosome pathway (ko04142), the intestinal immune network for IgA production (ko04672), and the phagosome pathway (ko04145) for each pairwise comparison are shown in Figure 9. Compared with the PBS group, *A. hydrophila* infection markedly upregulated the expression of genes in lysosomal components (LGMN, TPP1, GALC, GUSB, LAMAN, NEU1, GNS, and LYRLA3), membrane transporters (ABCA2), and associated factors (saposin, CLN5, MCOLN1, and LITAF family members), as well as phagosomal components (Rab5/7, RILP, v-ATPase, CALR, and calnexin) and an IgA-axis node (CXCR4) in the intestine of crucian carp. Supplementation with *L. sakei* JO12 markedly attenuated the *A. hydrophila*–induced upregulation of these intestinal transcripts, including genes associated with lysosomal components (LGMN, TPP1, GALC, GUSB, LAMAN, NEU1, GNS, LYRLA3, and LITAF) and phagosomal components (Rab5, RILP, and CALR). *L. sakei* JO35 exhibited the most pronounced restorative effect, promoting downregulation of genes associated with lysosomal (LAMP) and phagosomal (TAP, MHC II, and COLEC11) profiles. Moreover, *L. sakei* JO12, JO26, JO35, and LGG all increased the expression of multiple genes involved in IgA synthesis.

## 4 Discussion

*Aeromonas hydrophila* is among the most prevalent pathogens in aquaculture. Infection with *A. hydrophila* could disrupt the intestinal barrier of fish, precipitating dysbiosis of the gut microbiota, metabolic derangements, and ensuing systemic manifestations (36, 37). Antibiotic overuse in aquaculture has accelerated the emergence and dissemination of antimicrobial resistance and frequently disrupts host–microbiota homeostasis, with consequences for fish health and potential spillover risks to humans via the food chain (38, 39). In this study, effect of three *L. sakei* strains (JO12, JO26, JO35) and LGG on *A. hydrophila*-infected crucian carp was determined. Growth, histology, serum and intestinal biochemical indices, cytokine profiles, 16S rRNA sequencing, and intestinal transcriptomics of crucian carp were measured. LGG was used as a positive control strain in this article. Our multidimensional design aligns with recent calls for mechanistic probiotic evaluation across host, microbiome, and molecular layers in aquatic species, moving beyond growth-only endpoints and toward causal axes of mucosal protection and immunometabolic reprogramming (40, 41).

First, *in vitro* antagonism established that all three *L. sakei* strains produced larger inhibition zones than LGG against *A. hydrophila*, suggesting a robust capacity to secrete antimicrobial metabolites and/or competitively interfere with pathogen growth (Table 1). Although Oxford cup assays do not fully recapitulate intestinal conditions (pH, bile, mucus, microbe–microbe interactions), these results justified *in vivo* testing. Following this, experimental diets supplemented with *L. sakei* JO12, JO26, and JO35, and with LGG, according to established feed preparation protocols were formulated. Fish were randomly allocated to the respective diet groups, with a commercial feed group serving as the control. Body length and body weight were recorded at baseline and at the end of the trial and growth performance was calculated using standard formulas (Table 2). *In vivo*, crucian carp receiving *L. sakei* JO35 displayed the highest weight gain rate and specific growth rate and the lowest feed conversion ratio. These findings indicate enhanced nutrient utilization and metabolic efficiency in the presence of *L. sakei*. These observations are consistent with recent *Litopenaeus vannamei* studies in which *Lactobacillus acidophilus* and *Bacillus subtilis* improved growth via pathogen suppression and metabolic optimization (42). Differences between strains in our study likely reflect genomic determinants (bacteriocins, EPS architecture, adhesins) and acid/bile tolerance influencing colonization and metabolite output (43).

At the mucosal interface, histology demonstrated that *A. hydrophila* provoked mucosal edema and leukocytic infiltration, consistent with barrier disruption and active inflammation. *L. sakei* JO12, JO26 and JO35 alleviated these lesions, preserving goblet cells and mucosal architecture, whereas LGG failed to confer comparable protection in this model. Biochemical markers supported these findings and infection elevated intestinal ACP and AKP, markers linked to inflammatory activation and membrane transport remodeling, respectively. *L. sakei* broadly reduced serum ACP, JO12 and JO35 increased intestinal lysozyme, a key effector of innate antibacterial defense. In intestine, JO35 enhanced lysozyme and ACP, suggesting a systemic facet to the probiotic response. This pattern mirrored recent reports that LGG enhanced mucin production and attenuate epithelial damage under pathogen challenge in fish (44). The comparatively weaker performance of LGG in our study may underscore host- and context-specificity of probiotic action (45).

TNF-*α*, IL-1β, IL-10, IFN-*γ*, and MyD88 were inflammation-related cytokines involved in innate immunity in the intestines of crucian carp (46, 47). *A. hydrophila* infection increased TNF-α and IL-1β and decreased IL-10 in the intestine of crucian carp. *L. sakei* JO12, JO26 and JO35 supplementation reversed these trends by lowering TNF-α and IL-1β across strains and elevating IL-10. MyD88 was a central adaptor for most Toll-like receptors and was reduced by all *L. sakei* strains in infected intestines of crucian carp. A mouse study reported that dietary administration of *Lactobacillus agilis* SNF7 reduced pro-inflammatory cytokines (TNF-α and IL-1β) and increased IL-10, suggesting a potential anti-inflammatory immunomodulatory capacity of *Lactobacillus strains* (48). However, extrapolation from mammals to teleost fish should be made cautiously, because mucosal immune organization and immunoglobulin isotypes differ substantially between species. Thus, mammalian findings are supportive but not directly transferable, and fish-specific mechanistic validation is required. In crucian carp, our result indicated a directionally similar shift toward controlled inflammation under *L. sakei* supplementation. In addition, feeding *Lactobacillus delbrueckii* ameliorated *A. hydrophila*–induced intestinal inflammation in crucian carp by downregulating the mRNA expression of IL-1β, IL-8, TNF-α, and NF-κB p65 (49). This was consistent with the findings in our study.

Microbiome analyses revealed ecological hallmarks of disease and recovery. *A. hydrophila* infection increased Proteobacteria and reduced Firmicutes in the intestine of crucian carp echoing dysbiosis signatures linked to oxygen/ROS-driven bloom of facultative anaerobes and barrier permeability (50, 51). *L. sakei* supplementation reversed Proteobacteria expansion and suppressed *Acinetobacter*, an opportunistic, antibiotic-resistant genus, and only *L. sakei* JO35, reduced *Bacteroidota* (52). This trajectorie parallel recent fish studies demonstrating probiotic-driven suppression of Proteobacteria blooms and enrichment of beneficial commensals, often tied to restoration of hypoxic niches and reduction of epithelial oxygenation (53). Increased *Latilactobacillus* abundance in the intestine of crucian carp under *L. sakei* JO12 and JO35 groups supports direct niche establishment, while broader increases in beneficial taxa (Rhizobium lineage) may reflect cross-feeding and redox normalization in the gut ecosystem (54). Notably, only *L. sakei* JO12 and JO35, but not JO26 or LGG, significantly increased the intestinal abundance of *Latilactobacillus* in *A. hydrophila*–infected crucian carp. This discrepancy likely reflects strain-specific differences in intestinal colonization capacity, including adhesion to mucus and epithelial surfaces, resistance to bile salts and low pH, and ecological competitiveness within the resident microbiota. The rarefaction and PCA results confirm adequate sampling depth and a probiotic-driven community trajectory away from the dysbiotic state.

Our transcriptomic analysis identified DEGs predominantly enriched in immune and mucosal defense pathways, notably lysosome (ko04142), phagosome (ko04145), and the intestinal immune network for IgA production (ko04672). These signatures align with canonical PRR–PAMP recognition and downstream transcriptional activation of antimicrobial programs, consistent with host responses to pathogenic stimuli (55, 56). *A. hydrophila* infection markedly upregulated multiple lysosomal hydrolases and accessory factors, but selectively reduced the expression of LAMP family members, which are critical for phagosome–lysosome fusion. *L. sakei* JO26 prominently influenced TLR/C-type lectin receptor signaling and xenobiotic/drug metabolism. *L. sakei* LGG impacted lysosome and nicotinamide pathways but with weaker phenotypic rescue in the intestine of crucian carp. Supplementation with *L. sakei* JO26, JO35 and LGG restored LAMP expression, suggesting recovery of endolysosomal competence and more efficient resolution of infection (57). Complement-related readouts further supported enhanced opsonophagocytosis: JO26 increased iC3b, reinforcing CR3/CR4-dependent uptake. THBS1 (TSP1) was upregulated across probiotic groups, indicating immunoregulatory effects via CD36/CD47 that may aid controlled inflammation and tissue repair (58). *L. sakei* JO35 elevated IgA-related signatures and restored pIgR expression, strengthening epithelial transcytosis and sIgA-mediated immune exclusion at the lumen (55). Although IGH decreased in the JO12 group, the overall enhancement of the IgA network and pIgR points to a targeted, low-inflammation containment strategy rather than nonspecific humoral amplification. This study has limitations and causality between specific microbial changes and immune reprogramming requires validation via gnotobiotic or microbiota-transfer experiments in the future. Combinatorial or synbiotic formulations that potentiate JO35’s benefits may be also tested.

In conclusion, dietary *L. sakei*—particularly strain JO35—enhances growth performance, stabilizes intestinal microbial ecosystems, and restores mucosal immune homeostasis in crucian carp challenged with *A. hydrophila*. By reducing pathogen burden, reinforcing IgA-mediated exclusion, and reactivating lysosome–phagosome competence while tempering excessive TLR–MyD88 signaling, *L. sakei* provides a biologically coherent, antibiotic-sparing strategy to prevent and control enteric infections in aquaculture. These findings support the incorporation of *L. sakei* JO35 into standardized feeding protocols in aquatic animal aquaculture as an antibiotic-sparing probiotic to improve health and growth while limiting antimicrobial use. *L. sakei* JO12 ranked as a close second, *L. sakei* JO26 displayed selective advantages (complement opsonization via iC3b), and LGG’s effects were context-dependent and generally weaker in this model. Future work should test causality using gnotobiotic carp, targeted inhibition of TLR–MyD88 and PPAR pathways, and loss-of-function approaches for pIgR/LAMP orthologs. It will also be important to employ comparative genomics and secretomics to delineate strain-specific functional determinants.

## Data Availability

The original contributions presented in the study are publicly available. This data can be found here: https://www.ncbi.nlm.nih.gov/bioproject/PRJNA1406500.
